# HNRNPU and architectural lncRNAs as nuclear tethers of epithelial state stability

**DOI:** 10.1038/s44321-026-00440-6

**Published:** 2026-05-04

**Authors:** Longlong Luo, George L Sen

**Affiliations:** 1 Dermatology and venereology, Department of Medical Sciences, Uppsala University, Uppsala, Sweden; 2 Department of Medical Biochemistry and Microbiology (IMBIM), Uppsala University, Uppsala, Sweden; 3 Department of Dermatology, Department of Cellular & Molecular Medicine, Division of Epithelial Biology, University of California, San Diego, CA USA

**Keywords:** RNA Biology, Skin

## Abstract

Skin biology is commonly framed through signaling pathways that reprogram transcription in response to inflammatory and environmental cues. Here, a complementary perspective is proposed: epidermal homeostasis and disease recurrence may also depend on the physical organization of the genome within the nucleus. HNRNPU/SAF-A has emerged as an RNA-dependent architectural factor that links RNA binding to chromatin topology, while architectural long non-coding RNAs provide precedents for how RNA can scaffold nuclear compartments and influence higher-order genome organization. Building on these concepts, the Epidermal Differentiation Complex is considered as a tractable epidermal locus in which RNA-dependent nuclear tethering may help stabilize barrier gene programs. This framework further suggests that chronic inflammation could remodel chromatin architecture in ways that persist after apparent resolution, generating a “structural scar” that biases future responses. Although this model remains hypothetical, it is now experimentally testable. By integrating architectural RNA biology with epidermal differentiation and inflammatory memory, this Perspective provides a roadmap for investigating how nuclear structure may contribute to epithelial state stability and how it may be altered in inflammatory skin disease.

## Abstract (web summary)

In this Perspective, G. Sen and L. Luo propose that RNA-dependent chromatin architecture influence gene programs in skin cells and create lasting “structural scars” after inflammation, influencing future responses.

## Graphical abstract

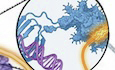

## Introduction: from informational signaling to architectural control in skin

Epithelial tissues are shaped by two requirements that can appear contradictory: the stability of lineage programs and plasticity under stress (Blanpain and Fuchs, 2014). The epidermis exemplifies this tension. Keratinocytes must maintain robust differentiation across time to enforce barrier integrity, yet adapt rapidly to injury, microbial challenge, or inflammation (Naik et al, 2017). The field has been remarkably productive in defining cytokine networks and immune–epithelial circuits that govern these adaptations (Pasparakis et al, 2014). Classical models of skin biology have successfully explained these responses through signaling cascades and transcription factor networks. However, these informational models do not fully explain how barrier gene programs remain robust over time, or why chronic inflammatory skin diseases such as psoriasis and atopic dermatitis often recur at the exact same anatomical sites after apparent clinical resolution.

One reason may be that these processes are not governed solely by informational signaling. Barrier programs are executed by coordinated gene clusters rather than isolated genes (Botchkarev et al, 2012; Mardaryev et al, 2014), and site-specific inflammatory recurrences imply that epidermal cells retain a localized memory of prior inflammation (Naik et al, 2017). While epigenetic mechanisms such as chromatin accessibility and histone modifications clearly contribute to this phenomenon, they ultimately operate within a physical three-dimensional (3D) genome scaffold (Larsen et al, 2021). This raises a complementary possibility: epithelial state stability and relapse are influenced not only by which regulatory factors are present, but by how chromatin is physically organized and constrained within the nucleus.

An architectural view treats the nucleus as a mechanically constrained 3D system in which chromatin loops, topologically associating domains (TADs), and nuclear compartments shape which genes can be accessed. Within this framework, RNAs are not merely downstream products of transcription; they can function as structural components that nucleate assemblies, stabilize protein networks, and couple chromatin to nuclear substructures (Quinodoz et al, 2021). Recent work has established the nuclear matrix protein HNRNPU (SAF-A) as an RNA-dependent architectural factor required for interphase chromosome organization and 3D genome topology (Fan et al, 2018; Marenda et al, 2022; Nozawa et al, 2017). Concurrently, a growing set of long non-coding RNAs (lncRNAs) has been shown to act as architectural scaffolds, with *XIST, NEAT1, NORAD, MALAT*1, and *FIRRE* providing rigorous mechanistic precedents (Colognori et al, 2019; Elguindy and Mendell, 2021; Hacisuleyman et al, 2014; Markaki et al, 2021; Shinn et al, 2025; Takakuwa et al, 2023).

**Figure 1:**
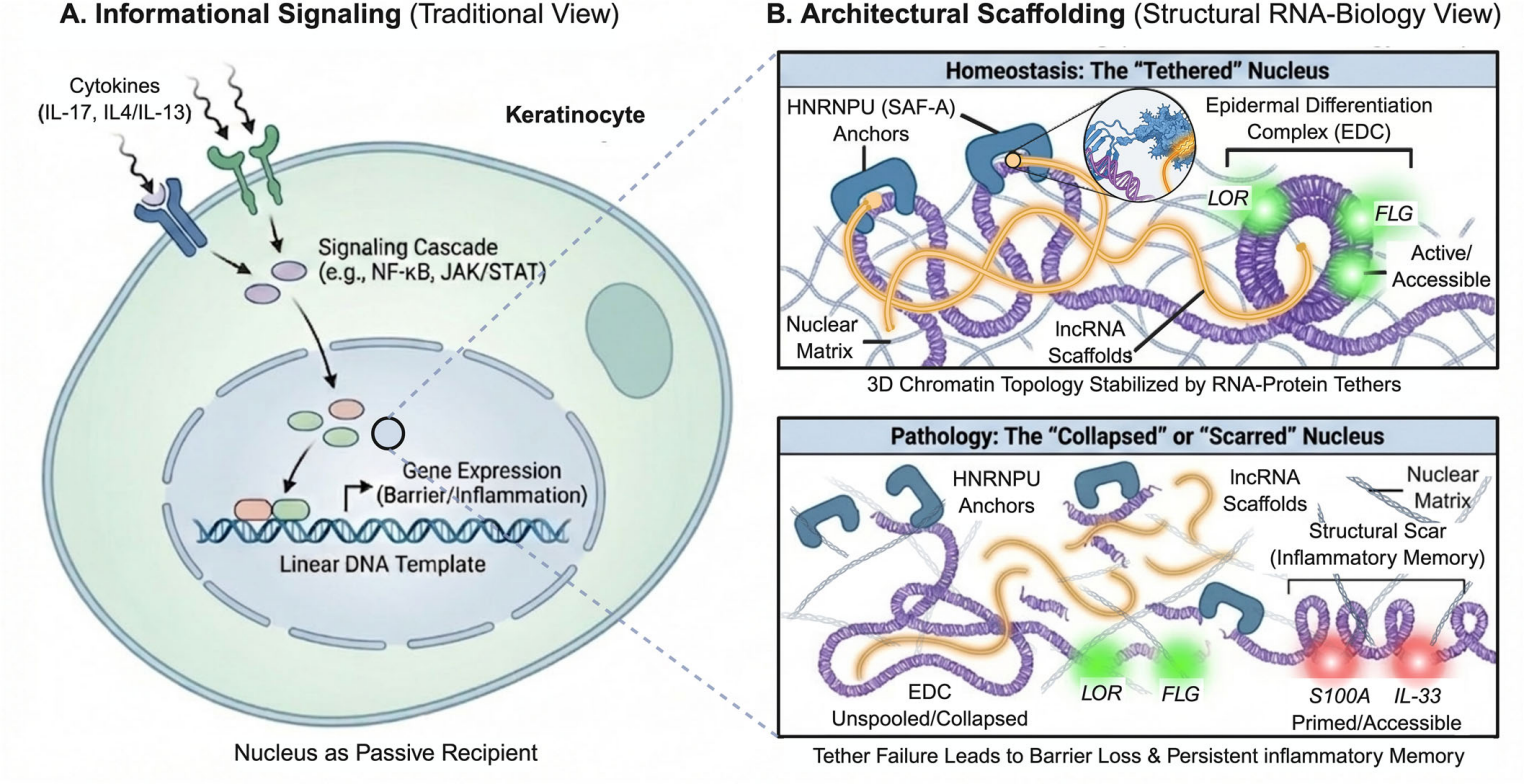
The architectural logic of the skin. (**A**) Informational view: Traditional models depict regulation as linear signaling cascades where cytokines drive transcription factors to bind a simplified DNA template. (**B**) Architectural view: Panel B depicts a hypothetical model proposed in this Perspective: HNRNPU (acting as “pins”) and architectural lncRNAs (acting as “wires”) physically tether chromatin loops, such as the Epidermal Differentiation Complex (EDC), to stabilize barrier programs within transcriptionally permissive nuclear compartments. Note the central positioning of the active EDC away from the nuclear periphery. In disease, disrupted tethering rewires genomic topology, creating a persistent “structural scar” that primes the tissue for inflammatory relapse. Green halos denote active, accessible barrier-gene neighborhoods, whereas the red halo denotes a primed, accessible inflammatory neighborhood associated with the proposed structural scar. Conceptual basis: SAF-A/HNRNPU is required for chromosome-scale organization and contributes to 3D genome topology; the EDC is regulated by long-range contacts and repositioned during differentiation; and nuclear scaffolds can stabilize primed chromatin loops (Fan et al, 2018; Ma et al, 2025; Nozawa et al, 2017; Poterlowicz et al, 2017; Quinodoz et al, 2021).

Here, we present a Perspective in which RNA-dependent nuclear tethering contributes to epidermal state stability. We propose that HNRNPU and specific architectural lncRNAs function as nuclear tethers to stabilize barrier-associated chromatin architecture, particularly at lineage-restricted loci such as the Epidermal Differentiation Complex (EDC). This contrast between classical informational signaling and the architectural framework proposed here is illustrated in Fig. 1A,B. We further hypothesize that chronic inflammation may remodel this nuclear topology in ways that bias future responses—a concept we refer to as a “structural scar” and depict schematically in Fig. 1B. Because this remains an emerging idea, the discussion below strictly distinguishes between established evidence, mechanistic inference, and explicitly testable hypotheses, and focuses on the experimental paths that could determine whether architectural RNA biology is a meaningful layer of epidermal regulation.

## HNRNPU as a mechanistic entry point for skin nuclear architecture

HNRNPU (also known as SAF-A) is a ubiquitously expressed RNA-binding protein that localizes primarily to the nucleus, where it regulates RNA–chromatin interactions and global genome architecture. Depletion of SAF-A disrupts interphase chromosome organization and alters chromosome territory behavior, establishing it as a large-scale genome folding factor rather than merely a local regulator of RNA metabolism (Nozawa et al, 2017). Genome-wide analyses further link HNRNPU to the maintenance of 3D genome topology, demonstrating that it is required for the proper strength of topologically associating domain (TAD) boundaries, specifically by stabilizing the binding of RAD21/cohesin to chromatin (Fan et al, 2018).

Mechanistically, HNRNPU acts as an architectural tether due to its modularity. Its N-terminal SAF-box enables direct DNA/chromatin engagement, while its C-terminal RGG/RG-rich domain binds structured RNAs (Kletzien et al, 2024a; Thandapani et al, 2013). The biological consequence of this modularity was elegantly demonstrated by Lawrence and colleagues, who showed that repeat-rich (C_0_T-1) RNAs function as scaffolds for chromosomal territories in interphase nuclei (Creamer et al, 2021). C_0_T-1 RNA interacts with SAF-A to maintain an open, decompacted chromatin structure that facilitates gene expression. Conversely, when C_0_T-1 RNA disintegrates from chromatin during mitosis or is depleted in senescence-associated heterochromatin foci, it leads to rapid chromosome compaction (Creamer et al, 2021). Furthermore, recent biophysical studies reveal that newly synthesized RNA interacts with SAF-A to form interconnected, tunable “microgels” that regulate local chromatin compaction (Marenda et al, 2024; Quinodoz et al, 2021). These microgels regulate chromatin compaction by modulating microphase separation, thereby opening transcriptionally active regions. Together, these established mechanisms show that HNRNPU functions as an ATP-dependent, RNA-guided molecular spring that physically holds DNA loops in accessible configurations.

HNRNPU is best understood within this broader context of nuclear architecture rather than as an isolated RNA-binding protein. In mammalian hepatocytes, *Hnrnpu* depletion causes a substantial expansion of lamina-associated domains and weakens TAD boundaries (Fan et al, 2018; Kadota et al, 2020). Those data suggest that without Hnrnpu acting as a structural anchor, the genome collapses toward the nuclear periphery (Fan et al, 2018). Crucially, chromatin topology relies on canonical architectural “hardware” such as CTCF and cohesin. Depletion of HNRNPU specifically weakens the binding of RAD21/cohesin to chromatin while CTCF binding remains relatively stable, indicating that HNRNPU provides a higher-order structural framework that stabilizes CTCF-cohesin loop anchors (Fan et al, 2018). In parallel, recent work has re-established the nuclear matrix as a mechanistically relevant scaffold capable of stabilizing primed regulatory chromatin loops (Ma et al, 2025). Importantly, this hardware integration extends to lineage-specific control; in the epidermis, the p63–CTCF axis mediates enhancer–promoter loop formation that is essential for cell-type-specific gene expression (Qu et al, 2019; Rubin et al, 2017). Within this landscape, HNRNPU can be framed as an RNA-dependent “adapter” that anchors these hardware-defined loops to nuclear scaffolds.

The relevance of this global architectural hardware to epidermal biology is supported by direct genetic evidence. We recently demonstrated that conditional, homozygous ablation of *Hnrnpu* in the mouse epidermis prevents stratification and epidermal appendage formation, leading to severe barrier defects and neonatal lethality (Hong et al, 2025). However, a notable discrepancy exists between this severe murine phenotype and human pathology. In humans, mutations in the *HNRNPU* gene typically cause early infantile epileptic encephalopathy and intellectual disability (Bramswig et al, 2017; Shimada et al, 2018), but do not present with obvious skin abnormalities. This divergence likely reflects a gene dosage effect: the severe murine phenotype results from a complete, tissue-specific knockout, whereas human patients are typically heterozygous (haploinsufficient). A complete loss of *HNRNPU* in humans is presumably embryonic lethal. Furthermore, it is highly likely that human keratinocytes possess redundant scaffolding mechanisms or compensatory hnRNP family members that buffer the epidermis against heterozygous loss.

**Figure 2:**
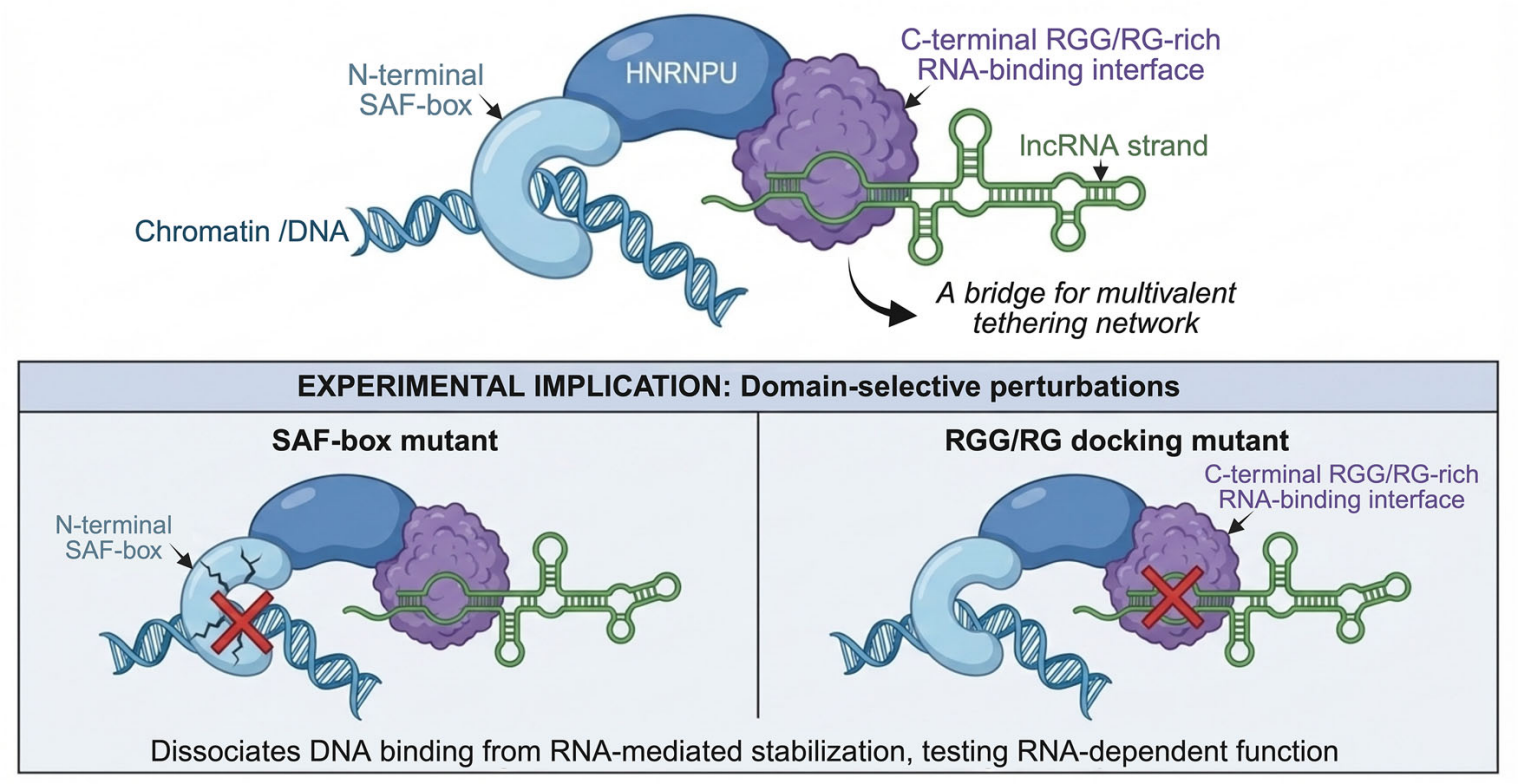
Micro-scale unit: the lncRNA–HNRNPU bridge. HNRNPU is depicted as a two-module tether: an N-terminal SAF-box that clamps chromatin and a C-terminal RGG/RG-rich RNA-binding interface that docks to structured RNA elements. The lncRNA strand threads through the RNA-binding interface, forming a bridge that can be repeated across many sites to build a multivalent tethering network. Key experimental implication: domain-selective perturbations (SAF-box versus RGG/RG docking mutants) should dissociate DNA binding from RNA-mediated stabilization of chromatin contacts, providing a direct test for RNA-dependent architectural function (Kletzien et al., 2024a, b; Kolpa et al., 2022).

Building on these established global functions, we hypothesize that the widespread scaffolding capacity of HNRNPU is locally co-opted in the epidermis to stabilize the 3D topology of specific barrier-critical gene clusters. We propose that structured, tissue-specific lncRNA elements dock onto HNRNPU’s RNA-binding interface while the SAF-box remains tethered to chromatin, forming a multivalent scaffolding network (Fig. 2). In this hypothetical model, RNA–protein interactions might maintain loop stability long after initiating inflammatory signals wane. However, because HNRNPU binds RNA degenerately, this model implies the existence of an additional regulatory layer that confers locus-specific targeting to the nuclear matrix. Before exploring the nature of these potential targeting factors, it is first essential to define a tractable epidermal locus where such architectural regulation can be directly observed and tested.

## The EDC as a locus-scale testbed for epidermal genome architecture

The Epidermal Differentiation Complex (EDC) provides the clearest locus-scale epidermal framework in which an architectural RNA hypothesis can be examined. Located on human chromosome 1q21, the EDC spans approximately 2 Mb and contains multiple barrier-associated gene families required for terminal keratinocyte differentiation and cornified envelope formation (Marenholz et al, 2001). Its biological importance lies not simply in gene density, but in the necessity for coordinated, high-level activation of many genes within a tightly defined developmental window (Botchkarev et al, 2012; Mardaryev et al, 2014; Poterlowicz et al, 2017). Such coordination is difficult to explain exclusively by promoter-centric transcriptional logic, pointing to a critical contribution from higher-order chromatin organization.

Direct evidence supports the view that the EDC is organized as a structured three-dimensional regulatory unit rather than a simple linear cluster of co-expressed genes. A high-resolution 5C analysis showed that the locus contains distinct chromatin interaction networks spanning both gene-rich and gene-poor topologically associating domains, with contacts occurring not only within individual TADs but also between adjacent domains (Poterlowicz et al, 2017). Crucially, the EDC is physically dynamic across epidermal differentiation. In progenitor keratinocytes, the locus is relatively compact and positioned toward the nuclear periphery. Upon differentiation, the locus undergoes profound topological remodeling. Rather than simply looping out of a chromosome territory, the EDC relocates into nuclear compartments enriched by SC-35 speckles, where active transcription is favored (Mardaryev et al, 2014). Because nuclear speckles contain non-coding RNAs, including *MALAT1* (Metastasis-Associated Lung Adenocarcinoma Transcript 1), that facilitate gene expression, this spatial relocation aligns conceptually with an RNA-dependent architectural framework. This process of EDC relocation from the nuclear periphery to the nuclear interior is, at least in part, regulated by the ATP-dependent chromatin remodeler Brg1. Simultaneously, the 3D conformation of the EDC in differentiating keratinocytes is governed by the chromatin architectural protein Satb1. Both Brg1 and Satb1 serve as direct downstream targets for the master epidermal transcription factor p63 (Fessing et al, 2011; Mardaryev et al, 2014). Consequently, in the homeostatic keratinocyte nucleus, the active EDC locus, including late differentiation genes such as LOR and FLG, is positioned more centrally and away from the nuclear periphery, as illustrated in Fig. 1B (upper panel).

These established features make the EDC an exceptionally useful experimental testbed: it is lineage-restricted, barrier-relevant, topologically organized, and already functionally connected to known epidermal architectural regulators (Brg1, Satb1, p63). However, it must be explicitly noted that there is currently no experimentally validated link between HNRNPU and the EDC complex. The structured EDC topology does not inherently prove that HNRNPU organizes this specific locus, nor does it establish that a specific lncRNA is required for its regulation.

Therefore, we highlight the EDC not as a proven target, but as a highly tractable locus in which the proposed RNA-dependent nuclear tethering model can be directly tested. It provides the necessary measurable outputs such as long-range contact frequency, nuclear repositioning, and coordinated expression of barrier genes to determine whether an additional RNA-dependent architectural layer contributes to epithelial state stability.

## Architectural lncRNAs as scaffolds: validated precedents and transferable principles

The present architectural model depends not only on HNRNPU acting as a global anchor, but also on the existence of tissue-specific RNAs capable of imparting spatial specificity to nuclear organization. “Nuclear architectural RNA” refers to RNA species whose functions are expressed in part through physical properties such as length, modular binding motifs, structured elements, and capacity for multivalent interactions rather than through coding potential (Hirose et al, 2025). While architectural RNAs can also operate in the cytoplasm, within the nucleus they act by (i) scaffolding ribonucleoprotein assemblies, (ii) promoting phase-separated condensates or compartmentalization, (iii) tethering chromatin to nuclear substructures, or (iv) stabilizing long-range chromatin contacts (Hirose et al, 2025; Kawaguchi and Hirose, 2012; Mattick et al, 2023).

A central biophysical principle of these scaffolds is multivalency: repeated low-to-moderate affinity interactions across many sites can yield high effective avidity and stable organization. This is particularly relevant to nuclear architecture, where proteins and RNAs often contain modular domains and intrinsically disordered regions capable of multiple weak interactions (Banani et al, 2017). Consequently, while disrupting a single RNA–protein interaction may have a limited effect, selectively disrupting a key domain that enables multivalent docking can collapse an architectural network.

To use the term “architectural lncRNA” rigorously (Engreitz et al, 2016), scaffold evidence must be defined operationally: an RNA must support physical nuclear organization that cannot be reduced simply to transcriptional output. Established criteria include domain-defined assembly, direct docking to chromatin or matrix proteins, and measurable topological changes upon RNA perturbation.

While candidate architectural RNAs in the epidermis remain to be fully characterized, several well-validated lncRNA archetypes in other systems provide essential mechanistic templates. The most stringent benchmark for RNA-guided chromatin restructuring is *XIST* (X-inactive specific transcript), which orchestrates chromosome-wide inactivation not merely by recruiting silencing machinery, but by nucleating local protein gradients that physically reshape chromatin state and positioning (Colognori et al, 2019; Markaki et al, 2021; Perotti et al, 2025).

A second archetype, *NEAT1* (Nuclear Paraspeckle Assembly Transcript 1), demonstrates how a single lncRNA can specify compartment formation (nuclear paraspeckles) through modular domains and selective protein recruitment (Hirose et al, 2019; Takakuwa et al, 2023). Crucially, *NEAT1* directly intersects with epidermal biology: it is a ΔNp63-regulated lncRNA that influences epidermal differentiation programs (Fierro et al, 2023), providing an established bridge between nuclear body assembly and epithelial state control. Similarly, *NORAD* (Non-Coding RNA Activated By DNA Damage) illustrates how an RNA can nucleate multivalent protein assemblies with consequences for genome stability (Elguindy and Mendell, 2021). *MALAT1* supports spatial organization within nuclear speckles and helps define RNA-rich microphases (Shinn et al, 2025; Tripathi et al, 2010). This is highly relevant to the epidermis, as the repositioning of the EDC into SC-35-enriched nuclear speckles during differentiation (discussed above) aligns perfectly with a *MALAT1*-like microenvironmental tethering mechanism.

Finally, *FIRRE* provides the most direct precedent for the model proposed here, as it connects an architectural lncRNA directly to HNRNPU (SAF-A). *FIRRE* contains a defined repeat domain that mediates HNRNPU binding and is required for maintaining spatial proximity among multiple associated chromosomal regions. Functionally, loss of *FIRRE* disrupts higher-order nuclear co-localization, while HNRNPU depletion phenocopies this loss of spatial proximity, supporting an established HNRNPU–lncRNA topological module (Hacisuleyman et al, 2014).

Collectively, these validated archetypes—*XIST*, *NEAT1*, *NORAD*, *MALAT1*, and *FIRRE*—establish a transferable biophysical logic: RNA-dependent regulation is constrained not simply by which transcription factors are present, but by whether RNA–protein assemblies can physically engage target chromatin in the correct nuclear microenvironment. While we hypothesize that analogous lncRNA-dependent tethering networks stabilize barrier loci such as the EDC, these archetypes demonstrate that the biophysical hardware required for such a mechanism already exists in nature.

## Perspective: RNA-dependent nuclear tethering as a mechanism of epidermal state stability

Having reviewed the established principles of nuclear architecture, we now propose the central hypothesis of this Perspective: epidermal state stability depends, in part, on RNA-dependent nuclear tethering. We propose that the epidermis provides a natural arena for this biology. Keratinocyte differentiation is coordinated across large genomic loci (e.g., EDC) and involves the concurrent activation of densely clustered gene families (Mardaryev et al, 2014). Because such coordination is difficult to explain exclusively through independent, linear promoter activation, we hypothesize that it relies on locus-scale regulatory neighborhoods supported by architectural stabilization. In this proposed model, barrier-associated loci are not maintained solely by transcription factor occupancy or local chromatin marks, but also by physical coupling to nuclear scaffolds through multivalent RNA–protein interactions. The distinction between the traditional informational model and the tethering model proposed here is summarized in Fig. 1A and B, respectively.

HNRNPU provides a plausible molecular anchor for such a mechanism because it combines chromatin association with RNA-binding capacity, while architectural lncRNAs offer a means of conferring locus specificity, multivalency, and compartmental engagement. Rather than acting as passive bystanders, these RNA–protein interactions may help stabilize the three-dimensional configurations that allow barrier programs to be executed reproducibly across repeated cycles of epidermal renewal.

A useful way to conceptualize this model is as a modular tethering system. HNRNPU can be viewed as an architectural bridge whose DNA-binding and RNA-binding functions are separable, allowing it in principle to connect chromatin loops to RNA-containing nuclear assemblies. In homeostatic epidermis, such tethering could help maintain the EDC and other lineage-restricted loci in a configuration permissive for coordinated barrier gene expression. Importantly, this would not replace classical signaling logic. Rather, it would provide the structural context within which signaling pathways are interpreted, much as enhancer–promoter communication depends not only on transcription factors but also on the spatial organization of the underlying chromatin.

Within this framework, architectural lncRNAs need not act as universal master regulators. A more plausible scenario is that a subset of nuclear-retained RNAs provides positional or compartmental specificity to a broadly acting architectural factor such as HNRNPU. Translated to the epidermis, this suggests that one or more keratinocyte-enriched lncRNAs may help recruit, stabilize, or spatially restrict HNRNPU-containing tethering complexes at barrier-relevant chromatin neighborhoods.

The strength of this Perspective lies in its falsifiability. If the model is correct, perturbing HNRNPU or disrupting candidate architectural lncRNAs should change not only gene expression, but also locus topology and nuclear positioning. The value of proposing RNA-dependent nuclear tethering in the epidermis is therefore that it generates a coherent set of experiments capable of distinguishing structural contributions from mere transcriptional consequences.

## From inflammatory memory to architectural memory

Barrier epithelia do not simply revert to a naïve baseline after inflammation. Instead, accumulating evidence indicates that prior inflammatory exposures establish a durable “memory” state, manifesting as altered responsiveness, accelerated recall programs, and long-lived tissue adaptation. In the skin, this concept is well-established; inflammatory memory involves persistent enhancer accessibility and altered chromatin states that accelerate transcriptional reactivation upon restimulation (Larsen et al, 2021; Naik et al, 2017; Niec et al, 2021). These classical epigenetic mechanisms are the default explanation for long-lived epithelial recall and help explain relapse-like phenomena in chronic inflammatory skin diseases.

Building on this foundation, we propose a narrower hypothesis: could a subset of this persistence be maintained through nuclear architecture? If inflammatory memory reflects durable changes in regulatory potential, we propose that some of that durability depends on sustained alterations in chromatin contacts, nuclear positioning, or compartmental engagement. We term this concept “architectural memory.” This is not an alternative to epigenetic memory, but a physical manifestation of it. In this view, inflammation-induced remodeling could change the probability that certain loci remain tethered to scaffold-associated microenvironments after inflammatory signals have waned, generating a persistent “structural scar” that primes the tissue for rapid maladaptive reactivation.

An important unresolved question is which epidermal cell populations might bear this hypothetical architectural memory. Because differentiated keratinocytes are short-lived, continuously shed, and immune-dampened (Liu et al, 2024), long-term architectural memory is most plausibly maintained in epidermal stem cells or long-lived basal keratinocytes, which can preserve regulatory states across tissue renewal. At the same time, more differentiated progenitors may still contribute to shorter-lived structural biases during active inflammation or early relapse. This distinction matters experimentally: if architectural memory exists, it will be important to determine whether it is a property of long-lived stem/progenitor compartments, broadly distributed basal keratinocytes, or transient inflammatory states in differentiating cells. Clarifying this will also help distinguish stable disease memory from more immediate structural responses to inflammation.

Current evidence from psoriasis and atopic dermatitis is compatible with this hypothesis, though it does not yet prove it (Tang et al, 2021; Xu et al, 2022). Disease-associated chromatin interaction studies have shown that keratinocyte regulatory wiring is dynamic and that inflammatory skin disease is associated with altered long-range contacts and epigenomic remodeling (Ray-Jones et al, 2020; Reynolds et al, 2021; Sahlén et al, 2021; Shi et al, 2021). These findings support the idea that disease states are accompanied by non-random changes in three-dimensional regulatory organization (Sahlén et al, 2021; Shi et al, 2021). However, most current datasets compare disease versus non-disease states at single time points. To establish a structural scar in a strict mechanistic sense, it remains necessary to demonstrate that these specific 3D topological changes persist after clinical resolution. Therefore, while inflammatory memory in barrier epithelia is an established fact, architectural memory in the epidermis remains a testable hypothesis.

Taken together, the available data support a cautious but productive conclusion: inflammatory memory in barrier epithelia is established; architectural memory in epidermis remains a hypothesis. The rationale for proposing it is not that current evidence already proves persistent structural scars, but that existing chromatin interaction data, together with the emerging biology of RNA-dependent nuclear scaffolds, make the idea experimentally tractable (Larsen et al, 2021). The next step is therefore not broader speculation, but longitudinal and cell-type-resolved testing of whether inflammatory resolution leaves behind persistent changes in locus positioning, chromatin contacts, or scaffold engagement that bias future epidermal responses.

## Experimental roadmap: decisive tests of the model

The value of the present Perspective lies in its falsifiability. If RNA-dependent nuclear tethering contributes to epidermal state stability, then perturbing the system should alter not only transcriptional output, but also the physical organization of chromatin. The most informative experiments will therefore be those that connect molecular perturbation, locus architecture, and functional epidermal phenotype in the same framework. A central goal of the roadmap is to distinguish global architectural disruption from locus-specific tethering effects, particularly at the Epidermal Differentiation Complex (EDC) (Poterlowicz et al, 2017).

A first decisive question is whether HNRNPU stabilizes EDC topology. This can be tested by acute HNRNPU perturbation in keratinocytes followed by locus-resolved and genome-wide conformation assays, including Micro-C/Hi-C class approaches and targeted Capture-C/4C across the EDC (Kempfer and Pombo, 2020; Ray-Jones et al, 2020; Shi et al, 2021). The logic here is straightforward: if HNRNPU contributes directly to EDC architecture, then perturbation should alter long-range contact frequencies, enhancer–promoter connectivity, and potentially nuclear positioning of the locus. Acute rather than chronic perturbation will be especially important because it minimizes secondary effects arising from differentiation failure or broad cellular collapse. In this setting, concordance between architectural change and barrier gene output would provide stronger evidence than either measurement alone.

A second decisive question is which RNAs engage HNRNPU in keratinocytes. Cross-Linking ImmunoPrecipitation (CLIP)-based approaches can define direct RNA partners, while RNA-centric capture strategies (e.g., ChIRP/CHART/RAP) can prioritize candidate nuclear-retained RNAs associated with chromatin and HNRNPU-containing complexes (Li and Fu, 2019). Candidate selection should be guided not only by differential expression, but also by nuclear localization, semi-extractability, structured RNA features, and positional enrichment near barrier-related loci. This step is critical because the current model does not require a universal epidermal scaffold RNA; it only requires that one or more candidate RNAs confer positional or compartmental specificity to a broadly acting architectural factor like HNRNPU. Identifying such RNAs would convert the current hypothesis from a locus-level model into a directly testable RNA–protein mechanism.

A third decisive test is domain separation. One of the most attractive aspects of HNRNPU as a candidate tether is that its chromatin-binding and RNA-binding functions are mechanistically separable. Perturbations targeting the SAF-box should compromise chromatin association, whereas perturbations affecting RGG/RG-mediated RNA docking should selectively impair RNA-dependent stabilization while preserving basal chromatin engagement (Kletzien et al, 2024a; Kletzien et al, 2024b). The strongest evidence for RNA-dependent tethering would come from experiments in which RNA-docking mutants disrupt locus topology or memory-like persistence more strongly than they disrupt basal chromatin association.

A fourth major question concerns cellular memory: establishing the structural scar longitudinally in the correct cell populations. As established earlier, long-lived structural memory is unlikely to reside primarily in short-lived differentiated keratinocytes. Instead, the most plausible candidates are epidermal stem cells and long-lived basal keratinocytes, although transient architectural states in differentiating progenitors may also contribute during active inflammation or early relapse. Resolving this will require longitudinal sampling across disease initiation, clinical resolution, and rechallenge, ideally in a cell-type-resolved framework combining single-cell transcriptomic and chromatin accessibility data with locus-resolved architectural measurements. Such experiments would directly test whether inflammatory resolution leaves behind persistent changes in topology or nuclear positioning in the compartments most likely to drive future epidermal responses.

Finally, the most convincing validation of the model will require integrating architecture with function. Structural changes should not be interpreted in isolation: the strongest case for RNA-dependent tethering would be one in which perturbation alters chromatin contacts, shifts nuclear positioning, changes coordinated expresion of barrier genes, and modifies the persistence or recall of inflammatory states. Conversely, if transcription changes occur without measurable architectural consequences, then the model would need to be revised toward a more conventional RNA-regulatory explanation. This is why the roadmap proposed here is intentionally stringent. Its purpose is not simply to accumulate correlative evidence, but to define the minimal experimental framework capable of determining whether epidermal state stability is governed in part by RNA-dependent nuclear architecture.

## Conclusions and outlook

This Perspective argues that epidermal state stability depends, in part, on RNA-dependent nuclear architecture. In this proposed framework, HNRNPU/SAF-A provides a strong mechanistic anchor because it seamlessly links RNA-binding capacity to higher-order genome organization. Simultaneously, architectural lncRNAs offer well-established precedents for how RNA can scaffold nuclear compartments, shape chromatin topology, and confer spatial specificity. By utilizing the EDC as an unusually strong locus-scale testbed, we motivate a model in which RNA-dependent nuclear tethering helps stabilize barrier-associated chromatin organization, thereby supporting robust and reproducible epidermal differentiation.

The central proposal advanced here is that chronic inflammation may perturb this architectural layer, generating a persistent “structural scar” that biases subsequent epidermal responses. This possibility is highly consistent with current concepts of inflammatory memory and with emerging disease-associated chromatin interaction datasets in psoriasis and atopic dermatitis. However, it must be emphasized that this mechanism is not yet established. The value of this model, therefore, lies not in claiming that architectural memory has already been proven in the skin, but in providing a falsifiable, experimentally tractable framework that connects molecular scaffold biology to locus-scale genome topology and persistent epithelial bias.

Looking forward, this framework suggests that epithelial relapse may not be governed solely by cytokine signaling or transcription factor networks, but also by the ability of the nucleus to preserve or re-establish specific 3D structural states. If validated, this would reposition structural RNA biology from a niche aspect of nuclear organization to a central principle of tissue homeostasis, inflammatory persistence, and barrier disease.

From a translational perspective, this raises the intriguing possibility of “architectural therapeutics”. If disease chronicity is rooted in a structural scar, future interventions might aim not simply to block acute inflammatory signals, but to “re-tether” the genome—perhaps utilizing antisense oligonucleotides (ASOs) or small molecules designed to disrupt maladaptive RNA–protein docking and restore healthy nuclear topology. The next challenge for the field is therefore clear: to determine whether RNA-dependent tethering is merely compatible with epidermal regulation, or whether it constitutes a previously unrecognized, targetable layer of control over epithelial identity and disease memory.

## Limitations and open questions

Several limitations and evidence gaps must be made explicit to maintain scientific rigor and guide future investigations.

First, global versus locus-specific architecture. As discussed, HNRNPU perturbation has profound global effects on chromosome organization (Nozawa et al, 2017; Poterlowicz et al, 2017). Because of this, it is highly possible that topological changes observed at loci like the EDC following HNRNPU depletion could be indirect consequences of global nuclear collapse or broad differentiation failure. At present, there is no experimentally validated, direct link between HNRNPU and the EDC complex. Therefore, future locus-level claims must be supported by assays that quantify topology at the EDC directly, using acute perturbation designs to uncouple local tethering from global structural decay.

Second, the missing epidermal lncRNA candidate and scaffold specificity. Currently, there is no identified candidate lncRNA in the epidermis proven to fulfill the proposed structural function. While rigorous archetypes exist (*XIST*, *NEAT1*, *FIRRE*), identifying a specific epidermal lncRNA that locally organizes barrier loci remains a critical missing piece of this model. Furthermore, many lncRNAs simply bind RNA-binding proteins without driving genome topology (Herman et al, 2022; Kopp and Mendell, 2018). Any future candidate lncRNAs in the epidermis will likely display pleiotropy, making it experimentally challenging—but absolutely necessary—to separate true architectural tethering effects from classical transcriptional regulation.

Third, hardware integration. Genome folding is physically shaped by cohesin, CTCF, active transcription, and nuclear compartments. Keratinocyte data demonstrate that the master regulator p63 can modulate chromatin loops via CTCF cooperation (Qu et al, 2019; Rubin et al, 2017). A key open question is whether RNA-dependent tethers (e.g., via HNRNPU) operate upstream of this loop hardware, downstream of it, or in parallel to stabilize existing loops.

Fourth, disease persistence and healing. Current psoriasis and AD datasets provide strong evidence for regulatory remodeling and disease-linked long-range contacts (Ray-Jones et al, 2020; Sahlén et al, 2021; Shi et al, 2021; Tang et al, 2021). However, most studies do not test topological persistence after clinical resolution. To establish a “structural scar” as a bona fide mechanism of inflammatory memory, longitudinal 3D topology measurements in matched lesional, non-lesional, and healed epidermis are strictly required.

Finally, computational inference and positioning. Deep learning analyses are beginning to formalize how RNA relates to chromatin organization (Kuang and Pollard, 2024). While these approaches can help prioritize epidermal lncRNA candidates, they must be grounded by rigorous biochemical perturbation experiments. Ultimately, structural RNA biology is not meant to replace cytokine- and immune-centric models of skin disease. Instead, it provides a complementary physical axis: the nuclear architecture, which determines how informational signals are interpreted and whether those transcriptional states revert or persist.

## Supplementary information

Peer Review File
