# Supplementary material for: HNRNPU and architectural lncRNAs as nuclear tethers of epithelial state stability
Source: EMBO Mol Med. 2026 May 4;18(6):2012–20. doi: 10.1038/s44321-026-00440-6 (PMC13270020; doi:10.1038/s44321-026-00440-6)
Supplement: Supplementary file 1 — Peer Review File [file 44321_2026_440_MOESM1_ESM.pdf]

EMBO Molecular Medicine

HNRNPU and Architectural lncRNAs as Nuclear Tethers of Epithelial State Stability

Longlong Luo and George Sen

Corresponding author: George Sen (gsen@health.ucsd.edu)

Review Timeline:

Submission Date:	30th Jan 26
Editorial Decision:	6th Mar 26
Revision Received:	17th Mar 26
Editorial Decision:	23rd Apr 26
Revision Received:	23rd Apr 26
Accepted:	24th Apr 26

Editor: Lise Roth

Transaction Report:

(Note: With the exception of the correction of typographical or spelling errors that could be a source of ambiguity, letters and reports are not edited. Depending on transfer agreements, referee reports obtained elsewhere may or may not be included in this compilation. Referee reports are anonymous unless the Referee chooses to sign their reports.)

6th Mar 2026

Dear Dr. Sen,

Thank you for submitting your article to EMBO Molecular Medicine. Please accept my apologies for the delay in responding, as one of the referees required additional time to complete their report. We have now received all three reports, and as you will see below, the referees found the article well written and the hypothesis interesting overall. However, they make several suggestions to improve the interest and impact of your work. We would therefore welcome a revised version of your manuscript that addresses these points.

In particular, we agree with the referees that the style of the article makes it better suited to being published as a 'Perspective', and we would therefore like you to rewrite it accordingly. According to our guidelines, Perspectives are scholarly reviews focused on specific areas of research with notable recent or prospective progress. They set the scene with recent developments and emphasise future directions. Perspectives may include a personal viewpoint but should prioritise evidence and testable hypotheses. Flexible in format, they typically include up to 5,000 words, 4 figures, and 50 references. For more information, see https://link.springer.com/journal/44321/submission-guidelines#cms-Article-types

Additionally, the distinction between established knowledge and your personal viewpoints or hypotheses should be clear.

Please attach a covering letter detailing how you have addressed each point raised by the referees.

For the figures, please note:
1. If there are certain aspects of your figure drafts that are based upon assumptions or where the scientific data remains ambiguous, please add a comment so that we can work with you on an accurate depiction. Please ensure the directionality and nature of interactions is presented accurately.
2. If the figures or part of the figures have been adapted from a published figure, please add this information to the figure legend (e.g., 'Adapted from...' or 'Based on...').
3. If you use an image data base for scientific iconography (e.g., BioRender), please let us know if you have a license that allows for publication in an academic journal.

Looking forward to receiving your revised manuscript at your earliest convenience.

With kind regards,

Lise Roth

Lise Roth, Ph.D
Senior Editor
EMBO Molecular Medicine

***** Reviewer's comments *****

Referee #1 (Remarks for Author):

Luo and Sen present a very interesting hypothesis. They propose a key role of the nuclear matrix protein HNRNPU (SAF-A) and some lncRNAs in the stabilization of gene clusters in the epidermis. They also propose that chronic inflammation rewires chromatin loops, thereby creating a topological memory. This may have important clinical implications. There are a few problems with the manuscript as detailed below.

The article starts with a good review on HNRNPU and structural lncRNAs, followed by the hypothesis and an outline of possible experiments that should be done to test the hypothesis. Therefore, it is not really a review, but rather a perspective.

Throughout the article, it should be more clearly mentioned what is known (and supported by citations) and what is the hypothesis of the authors. This is not always clear.

Although the hypothesis is exciting, the experimental evidence is so far rather weak. Mice with keratinocyte-specific knockout of Hnrnpu indeed have a major defect in skin development (impaired epidermal differentiation and stratification, resulting in impaired barrier function), but humans with mutations in the HNRNPU gene do not have obvious skin abnormalities. This should be discussed. In addition, information on the subcellular localization of HNRNPU in keratinocytes and its interaction partners is missing. There is also no candidate lncRNA, which may fulfil the proposed structural function and which - when depleted - induces epidermal differentiation abnormalities. Finally, there is so far no experimentally validated link between HNRNPU and the EDC complex.

3.1-3.4 is a nice review on HNRNPU, but if the major goal of the article is the description of the hypothesis, this part could be shortened. This is also the case for 5.1.1. - 5.1.4.

Various statements are not supported by references. For example, on page 6, 5th paragraph, it is mentioned that "Hnrnpu is required for the proper strength of TAD boundaries", but there is no reference. On page 13 it is mentioned that "there are indications of an additional epigenetic memory in keratinocytes", but there is no reference. These are only two of several examples.

"In a Nutshell" / Key points: We identify the nuclear matrix protein...This formulation is misleading, because the paper does not include any experiments. Identify should be replaced by propose.

Gene names should be in italics - this is important to clearly discriminate the gene from the encoded RNA and protein.

Page 12, last paragraph: Psoriasis and atopic dermatitis are not the most common chronic inflammatory diseases (cardiovascular diseases and joint diseases are more common) - they are the most common chronic inflammatory SKIN diseases.

Fig. 1B: It should be indicated in the figure that B is hypothetical (proposed by the authors).

Referee #2 (Remarks for Author):

This is an excellent review summarizing very important information on interaction of nuclear matrix protein HNRNPU with long non-coding RNAs in regulation of lineage-specific gene expression. Using keratinocyte-specific Epidermal Differentiation Complex locus as a model, the authors propose a hypothesis that in homeostatic conditions lineage-specific gene loci are stabilized by RNA-dependent tethering of chromatin loops to nuclear matrix. The authors develop further their hypothesis and propose that chronic inflammation disrupts homeostatic chromatin loop organization and leads to the formation of disease-linked long-range chromatin interactions associated with alterations in gene expression.
The review is nicely written and illustrated. However, there are few issues for the authors to consider:
1. There is Jeanne Lawrence's paper (Mol Cell, 2021, 81, 3509) demonstrating that repeat-rich (C0T-1) RNA forms a scaffold for chromosomal territories in interphase nuclei. (C0T-1) RNA interacts with SAF-A to form an open chromatin structure and facilitate gene expression. During mitosis (C0T-1) RNA is disintegrated from chromatin, while senescence-associated heterochromatin foci are depleted of (C0T-1) RNA leading to chromosome compaction. The authors need to cite and discuss this paper in the context of their hypothesis.
2. The authors need to consider including some clarification and additional information into their chapter regarding the EDC topology and conformation in homeostatic epidermal keratinocytes:
- Page 9, second paragraph. Please, change "loop out of chromosome territory" to "relocate into nuclear compartment enriched by SC-35 speckles". Because nuclear speckles contain non-coding RNAs including MALAT1 facilitating gene expression, this clarification would fit nicely into the author's hypothesis.
- The process of EDC relocation from nuclear periphery to nuclear interior is, at least in part, regulated by ATP-dependent chromatin remodeler Brg1, while EDC 3D conformation in differentiating keratinocytes is regulated by chromatin architectural protein Satb1. Both Brg1 and Satb1 serve as targets for p63 in keratinocytes (Fessing et al, 2012, Mardaryev et al, 2014).
3. Please, consider making changes in the Figure 1 B: in homeostatic keratinocyte nucleus, EDC locus including Lor and Flg genes is located more centrally and quite apart from the nuclear membrane (Mardaryev et al, 2014).

Referee #3 (Remarks for Author):

The review article by Luo and Sen describes the concept of HNRNPU-mediated structural functions of lncRNA in epidermal differentiation.
This is a timely and comprehensive review with interesting insights. However, the review is not easy to read for people without prior knowledge of protein-lncRNA as nuclear tethers. A few suggestions may increase readers' interest and ability to focus on reading through the article:

1. The whole article reads like a Perspective throughout. Sometimes, it is unclear, if one is reading the results of cited articles or the authors' own interpretations. One option is to dial down interpretations/insights in earlier sections and consolidate them to the latter or final sections.
2. The organization of the sections can also be improved to increase clarity.
The authors described "The review outline highlights a key gap: whether HNRNPU is required for EDC organization." Section 4.4-4.5 are speculative. Perhaps, move them after the "Exemplars of architectural lncRNAs" have been described or consolidate them in Section 7-working model.
3. Given the continuous renewal of epidermal cells, please discuss which cell populations, stem cells or basal keratinocytes should bear the "architectural memory" of EDC.
Minor:
1. When disease relevance is mentioned, please include the names of relevant diseases (e.g Page 3).
2. Some acronyms (e.g caRNA) are not explained, making it difficult to understand. An acronym/abbreviation section will be helpful.
3. Section 2-"Architectural RNA can act by "...." Besides nuclear functions, don't architectural RNAs also operate in the cytoplasm? "Nuclear Architectural RNA" maybe more proper here?

Point-by-Point Response to Referees

Manuscript ID: EMM-2026-23395P
Title: HNRNPU and Architectural lncRNAs as Nuclear Tethers of Epithelial State Stability

We thank the referees for their thoughtful and constructive comments, which have significantly improved the focus, rigor, and clarity of our manuscript. Below, we provide point-by-point responses to each referee's critiques.

Referee #1

1. The manuscript reads more like a Perspective than a Review.
Response: We agree and have rewritten the article accordingly as a Perspective, with a clearer focus on the central model, its implications, and experimental testability. The more review-like sections have been shortened substantially.

2. The distinction between known facts and the authors' hypothesis is not always clear.
Response: We agree and have clarified this throughout. The Introduction now explicitly states the perspective and its scope, and the revised manuscript more clearly distinguishes what is established, what is inferred, and what is proposed.

3. The experimental evidence remains limited; there is no validated HNRNPU–EDC link, no clear epidermal lncRNA partner, limited keratinocyte localization/interaction data, and no obvious human skin phenotype.
Response: We appreciate this important point and have revised the manuscript to acknowledge these limitations more explicitly. We now state clearly that:

- The epidermal *Hnrnpu* knockout establishes biological necessity in skin, but not a locus-specific mechanism.
- The manuscript does not claim a validated HNRNPU–EDC interaction.
- No epidermis-specific architectural lncRNA has yet been established as an HNRNPU partner.
- Information on HNRNPU localization and interaction partners in keratinocytes remains limited.
- Obvious skin abnormalities have not been prominently described in human *HNRNPU*-mutant syndromes, discussing the likely gene-dosage effects between mouse models and human patients.
  These limitations are now stated directly in the revised text (Section 9: Limitations and open questions).

4. The HNRNPU and lncRNA exemplar sections are too long for a hypothesis-driven article.
Response: We agree and have shortened both sections substantially, retaining only the most relevant mechanistic points needed to support the Perspective.

5. Several statements lacked references.
Response: We have carefully rechecked the manuscript, added missing references (e.g., regarding TAD boundaries and inflammatory memory), and softened a number of statements where direct support was insufficient.

6. “We identify ...” in the key points is misleading for a non-experimental article.
Response: We agree and have revised the “In a Nutshell” section accordingly. It now uses Perspective-appropriate wording (e.g., "We highlight...", "This Perspective proposes...") rather than implying experimental discovery.

7. Gene names should be italicized.
Response: Corrected throughout to clearly discriminate genes and non-coding RNAs from proteins.

8. Psoriasis and atopic dermatitis are among the most common chronic inflammatory skin diseases, not chronic inflammatory diseases overall.
Response: Corrected.

9. Figure 1B should be indicated as hypothetical.
Response: We agree and have revised the figure and legend to make clear that Panel B represents a proposed hypothetical model.

Referee #2

1. Please cite and discuss the Jeanne Lawrence Mol Cell 2021 paper on C0T-1 RNA.
Response: We thank the referee for this suggestion and have incorporated the Lawrence *et al.* study into the HNRNPU / nuclear architecture discussion (Section 2). This strengthens the RNA-dependent architectural framework of the manuscript.

2. Please clarify EDC relocation and include Brg1, Satb1, and p63.
Response: We have revised the EDC section accordingly (Section 3). The manuscript now describes differentiation-associated relocation of the EDC toward an SC-35-speckle-enriched nuclear environment, and includes discussion of Brg1, Satb1, and their relationship to p63-dependent epidermal lineage control.

3. Figure 1B should better reflect more central positioning of the EDC in homeostatic/differentiating keratinocytes.
Response: We agree and have revised Figure 1B and its legend to reflect the central positioning of the active EDC locus (including the *LOR* and *FLG* genes).

Referee #3

1. The manuscript reads like a Perspective already; it is sometimes unclear what is evidence versus interpretation.
Response: We agree. In the revised manuscript, earlier sections are more concise and evidence-focused, while the interpretive framework is now concentrated in the later sections, particularly the central Perspective, architectural memory, and experimental roadmap sections.

2. The section organization could be improved, with speculative content moved later.
Response: We agree and have reorganized the manuscript substantially to ensure facts precede hypotheses. The revised structure is now:

1. Introduction: From informational signaling to architectural control in skin
2. HNRNPU as a mechanistic entry point for skin nuclear architecture
3. The EDC as a locus-scale testbed for epidermal genome architecture
4. Architectural lncRNAs as scaffolds: validated precedents and transferable principles
5. Perspective: RNA-dependent nuclear tethering as a mechanism of epidermal state stability
6. From “inflammatory memory” to “architectural memory”
7. Experimental roadmap: decisive tests of the model
8. Conclusions and outlook
9. Limitations and open questions

3. Please discuss which epidermal cell populations might bear “architectural memory.”
Response: We have now addressed this explicitly in Section 6, proposing that long-term architectural memory is most plausibly maintained in epidermal stem cells and/or long-lived basal keratinocytes, while more differentiated progenitors may contribute to shorter-lived structural bias.

Minor 1. Disease relevance should name specific diseases.
Response: We have revised the text to refer explicitly to psoriasis and atopic dermatitis where relevant.

Minor 2. Some acronyms are not explained.
Response: We have improved acronym definition and terminology throughout to make the manuscript more accessible.

Minor 3. “Architectural RNA” may be too broad if the manuscript focuses on nuclear functions.
Response: We agree and have clarified that the manuscript is specifically focused on "nuclear architectural RNA" functions in epidermal biology.

23rd Apr 2026

Dear Dr. Sen,

Thank you for submitting your revised article to EMBO Molecular Medicine. Please accept my apologies for the late response as I was waiting for a referee report. Referees #1 and #2 evaluated your revised manuscript, and as you will see below, they are satisfied with the revisions. I will thus be able to accept your Perspective once the following editorial matters are addressed:

1. Please remove the sections "In a Nutshell" and "Data Availability"
2. Remove the heading "Declarations"
3. Rename "Conflict of Interest" to "Disclosure and Competing Interests Statement"
4. Remove "Author contributions" from the manuscript and provide CRediT (Contributor Role Taxonomy) terms in the submission system instead
5. Please move the Declaration of Generative AI to the Acknowledgements
6. Figure 1: please add callouts in the text to the individual panels A and B. For more clarity, please define the green vs. red halos in the legend.

Looking forward to receiving your revised manuscript at your earliest convenience.

With kind regards,

Lise Roth

Lise Roth, Ph.D
Senior Editor
EMBO Molecular Medicine

***** Reviewer's comments *****

Referee #1 (Remarks for Author):

The authors have very well responded to the comments of the reviewers, and the revised manuscript is much improved.

Referee #2 (Remarks for Author):

The authors addressed the reviewer's concerns and comments. Manuscript has been improved and might be accepted for publication.

The authors addressed the remaining editorial issues.

24th Apr 2026

Dear Dr. Sen,

I am pleased to inform you that your manuscript is accepted for publication and is now being sent to our publisher to be included in the next available issue of EMBO Molecular Medicine!

Your manuscript will be processed for publication by EMBO Press. It will be copy edited and you will receive page proofs prior to publication. Please note that you will be contacted by Springer Nature Author Services to complete licensing information. This Perspective article is free of charge.

If you have any questions, please do not hesitate to contact the Editorial Office.

Thank you for your interesting contribution to EMBO Molecular Medicine.

Sincerely,

Lise

Lise Roth, Ph.D
Senior Editor
EMBO Molecular Medicine
